# The first record of the monospecific genus *Rhinopalpa* (Lepidoptera: Nymphalidae) from China

**DOI:** 10.3897/BDJ.9.e70975

**Published:** 2021-08-24

**Authors:** Qiu-Ju He, Wen Shi, Chen-Yang Li, Chuan-Hui Yi, Zhuo-Heng Jiang, Shao-Ji Hu, Hui-Hong Zhang

**Affiliations:** 1 School of Biodiversity Conservation, Southwest Forestry University, Kunming, China School of Biodiversity Conservation, Southwest Forestry University Kunming China; 2 School of Life Sciences, Westlake University, Hangzhou, China School of Life Sciences, Westlake University Hangzhou China; 3 Yunnan Key Laboratory of International Rivers and Transboundary Eco-security, Kunming, China Yunnan Key Laboratory of International Rivers and Transboundary Eco-security Kunming China; 4 School of Agriculture, Yunnan University, Kunming, China School of Agriculture, Yunnan University Kunming China

**Keywords:** *
Rhinopalpa polynice
*, China, new record, Yuanjiang-Red River Valley, forestry species

## Abstract

**Background:**

The family Nymphalidae is the largest group of butterflies with high species richeness. *Rhinopalpapolynice* (Cramer, [1779]), a forest species, was discovered in the mid-stream of the Yuanjiang-Red River Valley of Yunnan Province for the first time, which represents the first record of the genus *Rhinopalpa* in China.

**New information:**

The species *R. polynice* (Cramer, [1779]) is the first record of the genus *Rhinopalpa* from China. The specimen was collected in the mid-stream of the Yuanjiang-Red River Valley of Yunnan Province. The female genitalia are described for the first time.

## Introduction

Nymphalidae is a cosmopolitan family of Papilionoidea with high species richness, which includes about 6,100 species in 12 subfamilies and 350 genera (Ackery et al. 1999, Hsu 2013, Wu and Hsu 2017). Most species are medium or large-sized and variable in wing shapes, colours and markings (Wu and Hsu 2017). Some species also exhibit strong seasonal phenotypes (Hsu 2013, Wu and Hsu 2017).

The genus *Rhinopalpa* C. & R. Felder, 1860 is placed in subfamily Nymphalinae (Savela 1990, Monastyrskii 2019, Inayoshi 2021). According to the molecular study including genus *Rhinopalpa*, this genus was regarded as Victoriini (Wahlberg et al. 2005 ,Chazot et al. 2019). The genus *Rhinopalpa* contains only one species, *Rhinopalpapolynice* (Cramer, [1779]) (Monastyrskii 2019), which is distributed widely from Indochina to Indonesia and the Philippines, with 10 subspecies described to date (Savela 1990, Inayoshi 2021). *Rhinopalpapolynice* has two subspecies in mainland SE Asia, ssp. eudoxia from Indochian to Malay Peninsula and ssp. birmana (Savela 1990, Osada et al. 1999, Monastyrskii 2019, Inayoshi 2021). *Rhinopalpapolynice* is a typical forests species and the larvae can develop successfully to adult on species of *Poikilospermum* (Cecropiaceae) and *Dendrocnide* (Urticaceae) in the wild (Monastyrskii 2019). Three specimens were recorded from the Red River Valley in North Vietnam (Monastyrskii 2019, Inayoshi 2021). However, there has been no record of this species in China.

In this study, a female *R. polynice* was collected from Yuanyang County, southeast Yunnan, China, which sits in the mid-stream of the Yuanjiang-Red River Valley and is isolated from the sites in North Vietnam where *R. polynice* was previously recorded. The female genitalia are described for the first time. The specimen, collected in this study, is the first record of the genus *Rhinopalpa* in China. Both specimen and dissected genitalia are deposited in the insect collection of Southwest Forestry University (SFU), Kunming, China.

## Materials and methods

Spread specimens were photographed by Canon 5DS (Canon, Japan) with medium grey background and the photos were adjusted using Adobe Photoshop CS (Adobe, USA).

To observe the female genitalia, the abdomen was treated with 1 ml 10% sodium hydroxide solution to digest soft tissue at 70℃ for 1 h and then dissected in a water-filled Petri dish under a stereoscope. The genitalia were then transferred to 80% glycerol for 12 h to render them transparent. A solutiion of 2% chlorazol black was used to dye the membranous parts for 10 min in order to obtain better photographic results. Photographs were taken with a Nikon SMZ1500 stereoscope (Nikon, Japan) and automatically stacked using Helicon Focus 7.5.8 (Helicon Software, USA). After observation and photography, a piece of card was cut, the genitalia were fixed to the card by white emulsion and pinned with the specimen to avoid confusion. The photographs were adjusted and arranged using Adobe Photoshop CS (Adobe, USA). Terminology of the female genitalia follows Hu et al. (2021).

## Taxon treatments

### Rhinopalpa polynice

(Cramer, [1779])

E0B18FEA-C7E3-5D41-B44C-DA0C61E125FD

#### Materials

- **Type status:**Other material. **Occurrence:** recordedBy: Wen Shi; individualCount: 1; sex: female; lifeStage: adult; disposition: in collection; **Taxon:** scientificName: *Rhinopalpapolynice* (Cramer, [1779]); kingdom: Animalia; phylum: Arthropoda; class: Insecta; order: Lepidoptera; family: Nymphalidae; genus: Rhinopalpa; specificEpithet: polynice; taxonRank: species; scientificNameAuthorship: (Cramer, [1779]); vernacularName: The Wizard; taxonomicStatus: accepted; **Location:** country: China; stateProvince: Yunnan Province; county: Yuanyang County; locality: Shalatuo Village; verbatimElevation: 928m; verbatimCoordinates: 23°6.047'N, 102°34.43'E; **Identification:** identifiedBy: Huihong Zhang; dateIdentified: 2020; **Event:** samplingProtocol: sweep net; year: 2020; month: 9; day: 29; habitat: Evergreen broad-leaved forest; **Record Level:** basisOfRecord: PreservedSpecimen

#### Description

**Figure 1.**
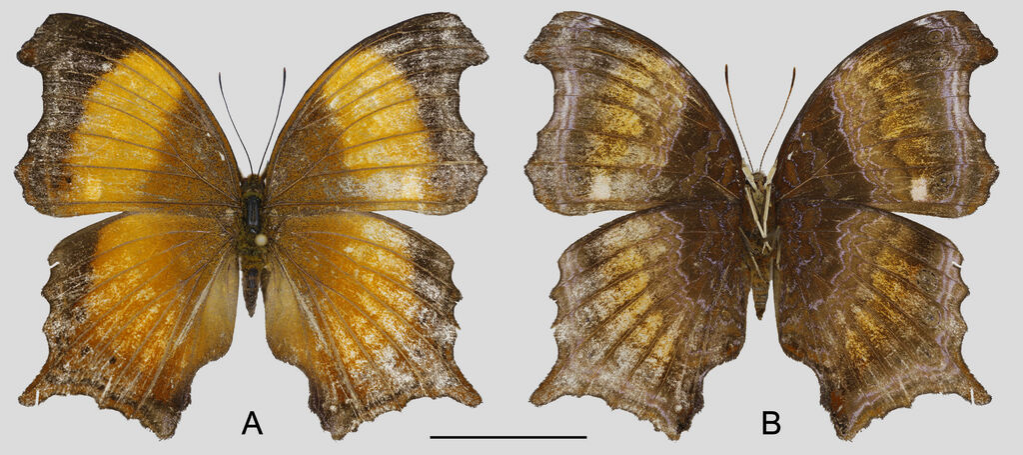
Female *Rhinopalpapolynice* (Cramer, [1779]) collected in Yuanyang County. **A.** upperside; **B.** underside; scale bar = 10 mm.

**Female** (Fig. 1): Forewing length 37 mm. Body brown covered with short hair dorsally, labial palpi brown, antenna straight and dark brown, legs greyish-yellow. Forewing: broad triangular with obvious zigzag termen, two angles at ends of veins M_1_ and CuA_2_, forming hook-shaped apex; upperside brownish in basal one third, discal area reddish-brown, postdiscal to termen area dark brown, space CuA_2_ with small subterminal black spot; underside dark brown, six fine purplish zigzag lines in brown basal area, six serial subterminal ocelli with purple pupil, brown iris and purple ring, terminal area with two parallel narrow purple wavy lines. Hindwing: square with obvious zigzag termen, acute short tail at end of vein M_2_; upperside with same colour configuration as forewing, three small subterminal black spots in spaces M1 to M_3_; underside with same colour configuration as forewing, including the purple lines in basal area, seven serial subterminal ocelli as those of forewing.

**Figure 2.**
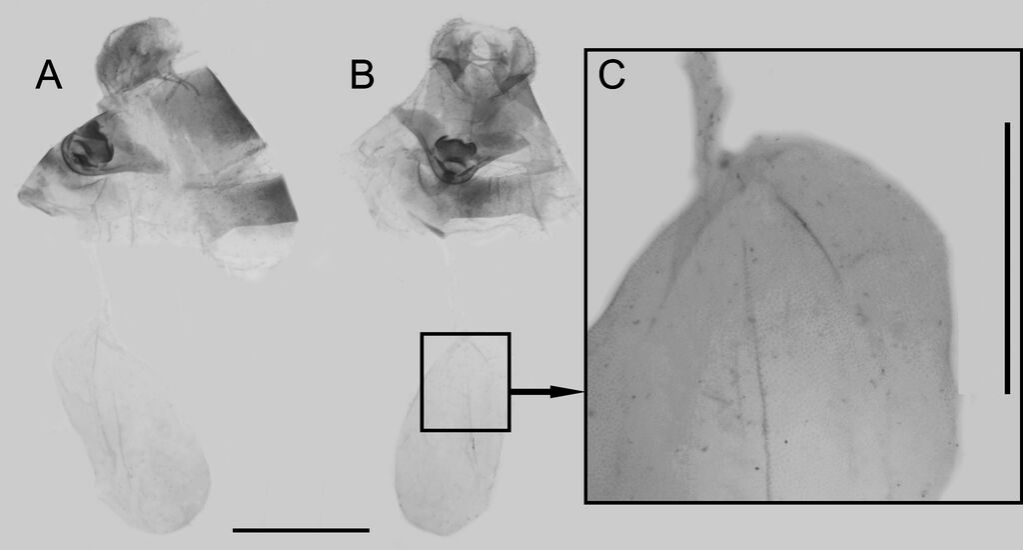
Female genitalia of *Rhinopalpapolynice* (Cramer, [1779]) collected in Yuanyang County. **A.** lateral view; **B.** ventral view; Scale bar = 5 mm **C.** signa enlarged, scale bar = 1 mm.

**Female genitalia** (Fig. 2): Papillae anales round and narrow. Lamella antevaginalis sclerotised and narrow, lamella postvaginalis sclerotised, with claw-like central part and two narrow lobes at both sides. Ductus bursae tubular and membranous, rather slender. Corpus bursae oval, signa comprised of two patches of tiny granules at both sides near base.

#### Distribution

**Figure 3.**
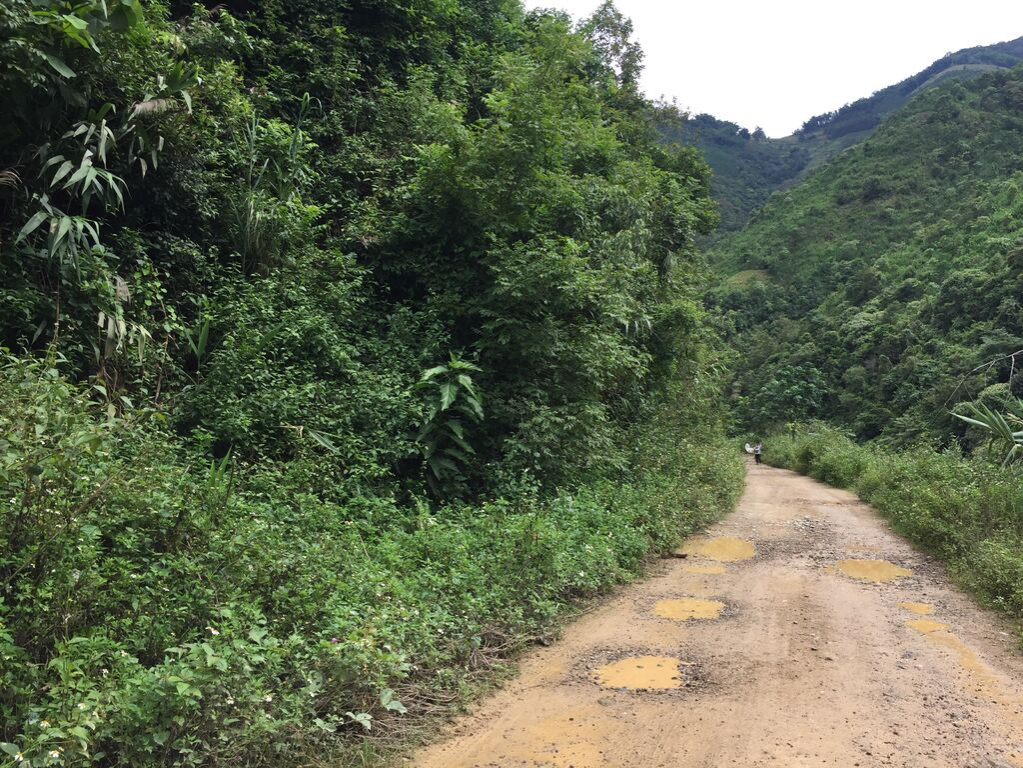
The habitat of *Rhinopalpapolynice* (Cramer, [1779]) in China: Shalatuo Village, Yuanyang County, Yunnan Province, SW China.

This species is currenly known from India, Myanmar, Laos, Vietnam, Thailand, Malaysia, Indonesia and Philippines (Savela 1990, Osada et al. 1999, Ek-Amnuay 2012, Monastyrskii 2019, Inayoshi 2021); and China is the new distribution area. (Fig. 3).

## Discussion

**Figure 4.**
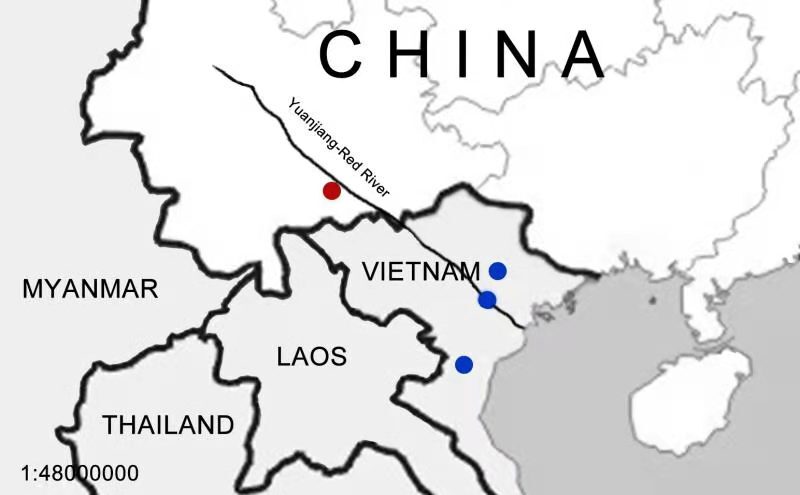
The distribution records of *Rhinopalapolynice* (Cramer, [1779]) from Yuanjiang-Red River Valley. Red circle: the new record in China; Blue circle: the records from Vietnam (Monastyrskii 2019, Inayoshi 2021).

China is a country with high biodiversity of butterflies with about 2020 species in recent records (Wu and Hsu 2017), especially in south Yunnan where the environment is highly heterogeneous (Zhang et al. 2020). In recent years, many new taxa and new records of butterflies were described and discovered (Hu and Zhang 2010, [Bibr B7292583][Bibr B7292592], Lang 2012, Lang 2017, Zhang et al. 2020). Most of these new records are forest species and were previously mainly recorded in Indochina (Fig. 4; Savela 1990, Osada et al. 1999, [Bibr B7292543][Bibr B7292670], Inayoshi 2021). As south Yunnan is adjacent to Indochina, it is logical that a number of Indochinese species could also be found in south Yunnan as the environment is similar (Li 1995, Chen et al. 2012). In light of this situation, the distribution range of these Indochinese species could be much wider than we know. Amongst these species, forest species like *R. polynice* are difficult to notice because of their particular habitat requirements and lower tolerance to human disturbance (Monastyrskii 2019).

In recent years, three cryptic species of butterflies were discovered from Indochina, Graphium (Pazala) daiyuanae Hu, Zhang & Cotton, 2018, Graphium (Pazala) wenlingae Hu, Cotton & Monastyrskii, 2019 and *Losariadoubledayi* (Wallace, 1865), all of them being forest species (Hu et al. 2018, Hu et al. 2019, Xu et al. 2020). Hence isolations are very likely between different populations in Indochina as a result of geographical barriers and environment heterogeneity, especially in forest species (e.g. the swampy grassland and savannah vegetation in the central part of Indochina). *Rhinopalapolynice* is a typical forest species living in lowland forests (Monastyrskii 2019), two subspecies of *R. polynice* are found in Indochina to Malay Peninsula, with ssp. eudoxia (type locality “Cote Malaye”) in South Thailand to Malay Peninsula and ssp. birmana (type locality “Lower Burmah”) in Assam, Myanmar and Indochina (Savela 1990, Osada et al. 1999, Monastyrskii 2019, Inayoshi 2021). *Rhinopalpap.birmana* is different from *R. p.eudoxia* by paler colour on the upperside, plus better-defined hindwing subterminal black spot in space M_1_ and narrower forewing subterminal band (Monastyrskii 2019). Monastyrskii (2019) treated the Vietnamese specimens as *R. p.birmana*. However, Inayoshi (2021) regarded all populations in Indochina and West Malaysia as *R. p.eudoxia*, due to insufficient morphological differences. In this study, as we only examined one specimen, it is impossible to further analyse its subspecies status with such limited material. Hence, the subspecies identity of *R. polynice* in Yunnan still requires future study.

## Supplementary Material

XML Treatment for Rhinopalpa polynice
